# Prevalence of diagnosed psychiatric disorders among adults who have experienced and internalized weight stigma

**DOI:** 10.1002/osp4.700

**Published:** 2023-07-25

**Authors:** Rebecca L. Pearl, Marian Hernandez, Caroline Bach, Laurie Groshon, Thomas A. Wadden

**Affiliations:** ^1^ Department of Clinical and Health Psychology College of Public Health and Health Professions University of Florida Gainesville Florida USA; ^2^ Department of Psychiatry Center for Weight and Eating Disorders Perelman School of Medicine at the University of Pennsylvania Philadelphia Pennsylvania USA; ^3^ Department of Psychology Georgia Southern University Statesboro Georgia USA

**Keywords:** anxiety, depression, eating disorders, psychiatric disorders, self‐stigma, weight stigma

## Abstract

**Objective:**

Experiences and internalization of weight stigma are associated with greater self‐reported psychological distress and symptoms of psychiatric disorders such as depression and anxiety. However, little is known about the extent to which individuals who have experienced and internalized weight stigma are diagnosed with or provided treatment for psychiatric conditions. The current study aimed to characterize the prevalence of diagnosed psychiatric disorders among adults with obesity who had experienced and internalized weight stigma.

**Methods:**

Weight‐loss treatment‐seeking adults with a history of experiencing weight stigma and high levels of internalized weight stigma were recruited for two clinical trials.

**Results:**

In Study 1 (*n* = 84, 83.3% women, 67.9% Black), 25% of participants reported a lifetime history of a mood disorder. Few participants (<10%) reported current psychiatric diagnoses or use of psychiatric medications. In Study 2 (*n* = 129, 88.4% women, 65.1% white), one‐third of participants reported a mood disorder history, and 21.7% reported an anxiety disorder history, with approximately 16%–18% reporting current diagnoses. In both studies, few participants reported a history of a diagnosed eating disorder despite high rates of current full‐ or subthreshold symptoms. Based on Beck Depression Inventory‐II scores, approximately 54%–64% of participants reported mild or greater symptoms of depression.

**Conclusions:**

Overall, lifetime history of diagnosed psychiatric disorders and current symptoms of depression and eating disorders were relatively high across two samples. More research is needed to determine the impact of weight stigma on the diagnosis and treatment of eating disorders and other psychiatric concerns.

## 1 INTRODUCTION

Individuals with a high body weight are frequently disparaged, discriminated against, and devalued by society (i.e., stigmatized).^1^ Media narratives about weight perpetuate negative stereotypes, promote idealization of lean bodies, and convey blame toward individuals in larger bodies.^2^ Exposure to negative weight messages begins at an early age, and weight is the leading reason for teasing and bullying among youth.^3^ Discrimination in education and employment disadvantages individuals with a high weight, with minimal protections or legal recourse when this discrimination occurs.^4^ Health care interactions are often fraught with judgmental or derogatory treatment by staff and providers of patients with a high weight, leading to poorer quality of care and to understandable avoidance of health care by patients.^5^ Altogether, experiencing weight stigma has been increasingly recognized as a form of chronic stress that adversely affects mental and physical health.^1^

Given the pervasiveness of weight‐stigmatizing messages and experiences, it is not surprising that some individuals with a high weight internalize negative weight stereotypes and attitudes, leading to self‐devaluation (i.e., self‐stigma).^6^ Estimates suggest that more than 50% of adults with obesity may internalize weight stigma to a high degree, with higher levels of internalization among women, white, young, and unmarried adults, and those with higher body mass indexes (BMIs) and less education.^7^ Some studies have identified other social factors that may increase the likelihood of internalizing weight stigma, such as: adverse childhood experiences^8^; younger age of onset of high weight and of weight stigma experiences^9^; and experiences of weight stigma from close family members or in work settings, especially those that result in high levels of distress.^10^ As with experiencing weight stigma from others, weight self‐stigma is linked to greater stress and poorer physical health outcomes.^11^ Despite increasing research on the topic of internalized weight stigma,^11^ few evidence‐based interventions exist to prevent or reduce it. Emerging work on internalized weight stigma interventions has primarily adapted evidence‐based approaches for addressing other mental health concerns (e.g., depression, anxiety) to specifically target responses to stigma and associated psychological distress.^12^, ^13^, ^14^, ^15^

A population‐based study from 2004 to 2005 found that more than half of adults with a body mass index (BMI) ≥25 kg/m^2^ who reported having experienced weight discrimination were diagnosed with a mood, anxiety, or substance use disorder.^16^ Other studies have found that experiencing or internalizing weight stigma is associated with more self‐reported symptoms of depression, anxiety, stress, and eating disorders.^17^ However, little is known about the prevalence of diagnosed psychiatric disorders among adults who have experienced and internalized weight stigma. Establishing the prevalence of clinically‐significant psychiatric concerns among persons most affected by weight stigma would provide important information about the mental health impact of weight stigma and the treatment needs of this population.

The current study examined the prevalence of diagnosed psychiatric disorders in two samples of weight‐loss treatment‐seeking adults with obesity who had experienced and internalized weight stigma. Psychiatric disorders may be particularly heightened in individuals who are seeking weight‐loss treatment, given prior evidence of psychological distress in this population.^18^ We hypothesized that participants would report a high prevalence of depression and anxiety disorders and a pronounced history of eating disorders.

## 2 METHODS

Participants were recruited for two randomized controlled trials that tested the effects of an intervention for internalized weight stigma in combination with and in comparison to behavioral weight loss treatment.^12^, ^19^

### 2.1 Participants

Participants were recruited from the community from April 2018 through March 2020. Key inclusion criteria were: age 18 years or older (upper limit = 65 years in Study 1); BMI ≥30 (or, in Study 2, BMI ≥27 with a comorbidity); report by interview of at least one lifetime experience of weight stigma (teasing, bullying, discrimination, or unfair treatment); and high levels of internalized weight stigma, as indicated by a score ≥4 on the Weight Bias Internalization Scale (WBIS)^6^ and confirmed by interview. Candidates were eligible to participate if they reported mild‐to‐moderate symptoms of depression, anxiety, or binge eating disorder (BED); individuals with *severe* symptoms were not eligible and were referred for appropriate treatment. Additional exclusion criteria included: current bulimia nervosa, substance use disorder, or thought disorder; current, active suicidal ideation or a suicide attempt in the past year; or a recent participation in weight‐related treatment (including psychotherapy). Due to effects on weight, individuals taking anti‐psychotic medications or certain anti‐depressant medications (e.g., bupropion) were excluded.

### 2.2 Procedures and measures

Study candidates were screened by telephone, followed by an in‐person behavioral evaluation with a psychologist. Participants provided informed consent before completing a medical screening; those who provided consent but were subsequently excluded from the trials due to medical eligibility (e.g., undiagnosed type 2 diabetes) were included in the current report. Both trials were approved by the institutional review board.

During the behavioral evaluation, candidates were asked by interview whether or not they had ever been diagnosed by a doctor or mental health professional with any of the psychiatric disorders listed in the DSM‐V (and if so, whether it was past or current). Participants were also assessed by interview for DSM‐V criteria of BED and night eating syndrome (NES). Participants were categorized as meeting full diagnostic criteria, or “subthreshold” criteria if symptoms were clinically significant but did not fully meet one criterion (e.g., binge episodes were not objectively large).^20^ Participants completed the Beck Depression Inventory‐II (BDI‐II), and cutoff scores were used to determine whether participants reported mild (scores 14–19), moderate (20–28), or severe symptoms of depression (≥29).^21^ Participants reported during the medical screening all current medications and the reason(s) they were prescribed.

### 2.3 Analyses

Descriptive statistics were computed for each sample to report the number and percentage of participants who: reported past or current psychiatric diagnosis; currently met full or subthreshold diagnostic criteria for BED and NES; had BDI‐II scores indicating mild, moderate, or severe symptoms of depression; and reported taking psychiatric medications. In posthoc exploratory analyses, data were combined across samples to test for differences by participant race in WBIS scores (using ANOVA) and in diagnosed mood and anxiety disorders, full or subthreshold BED, and use of psychiatric medications (using Pearson's chi‐square test).

## 3 RESULTS

### 3.1 Study 1

Study 1 included 84 participants who predominantly identified as Black or African American (Table 1). Mood disorders were the most frequently endorsed category of diagnosed disorders (25%), driven by major depressive disorder (22.6%; Table 2). Anxiety disorders and trauma or stressor‐related disorders affected approximately 8%–11% of participants. A small minority of participants reported that their diagnoses were current: less than 10% of participants reported current depression, and less than 5% reported current anxiety. Two participants reported a history of a diagnosed eating disorder.

**TABLE 1:** Sample characteristics.

Variables	Study 1	Study 2
	N (%) or M±SD	N (%) or M±SD
Age (years)	47.8 ± 11.2	50.0 ± 12.2
Gender		
Women	70 (83.3%)	114 (88.4%)
Men	14 (16.7%)	15 (11.6%)
Race		
Black or African American	57 (67.9%)	40 (31.0%)
White	24 (28.6%)	84 (65.1%)
Asian	2 (2.4%)	1 (0.8%)
American Indian or Alaska native	0	1 (0.8%)
Native Hawaiian or other Pacific Islander	0	0
Multiracial	N/A	3 (2.3%)
Unknown or not reported	1 (1.2%)	N/A
Ethnicity		
Hispanic or Latino	7 (8.3%)	5 (3.9%)
Not Hispanic or Latino	77 (91.7%)	124 (96.1%)
Four year college or more	43 (51.2%)	84 (65.1%)
Body Mass index (kg/m^2^)	39.2 ± 5.8	37.8 ± 5.8
Beck depression Inventory‐II	16.2 ± 8.0	14.6 ± 8.4

**TABLE 2:** Lifetime and current psychiatric diagnoses.

	Study 1		Study 2	
	N (%)	Current	N (%)	Current
Mood disorder	21 (25.0%)	8 (9.5%)	43 (33.3%)	23 (17.8%)
Bipolar disorder	0 (0%)		0 (0%)	
Major depressive disorder	19 (22.6%)	8 (9.5%)	40 (31.0%)	20 (15.5%)
Persistent depressive disorder	0 (0%)		0 (0%)	
Other	2 (2.4%)	0 (0%)	5 (3.9%)	3 (2.3%)
Psychotic or thought disorder	0 (0%)		0 (0%)	
Substance use disorder	3 (3.6%)	0 (0%)	2 (1.6%)	0 (0%)
Anxiety disorder	9 (10.7%)	4 (4.8%)	28 (21.7%)	21 (16.3%)
Panic disorder	1 (1.2%)	0 (0%)	7 (5.4%)	1 (0.8%)
Agoraphobia	0 (0%)		2 (1.6%)	0 (0%)
Social anxiety disorder	0 (0%)		1 (0.8%)	1 (0.8%)
Specific phobia	1 (1.2%)	1 (1.2%)	2 (1.6%)	1 (0.8%)
Generalized anxiety disorder	4 (4.8%)	2 (2.4%)	15 (11.6%)	13 (10.1%)
Other	3 (3.6%)	1 (1.2%)	9 (7.0%)	8 (6.2%)
Eating disorder	2 (2.4%)	0 (0%)	1 (0.8%)	0 (0%)
Anorexia nervosa	2 (2.4%)	0 (0%)	0 (0%)	
Bulimia nervosa	0 (0%)		1 (0.8%)	0 (0%)
Binge eating disorder	0 (0%)		0 (0%)	
Other	0 (0%)		0 (0%)	
Obsessive compulsive or related disorder	0 (0%)		1 (0.8%)	0 (0%)
Obsessive compulsive disorder	0 (0%)		0 (0%)	
Hoarding disorder	0 (0%)		0 (0%)	
Body dysmorphic disorder	0 (0%)		1 (0.8%)	0 (0%)
Trichotillomania	0 (0%)		0 (0%)	
Excoriation	0 (0%)		0 (0%)	
Other	0 (0%)		0 (0%)	
Sleep‐wake disorder	3 (3.6%)	0 (0%)	0 (0%)	
Insomnia disorder	2 (2.4%)	0 (0%)	0 (0%)	
Hypersomnolence disorder	0 (0%)		0 (0%)	
Other	1 (1.2%)	0 (0%)	0 (0%)	
Somatic symptom or related disorder	0 (0%)		0 (0%)	
Trauma‐ or stressor‐related disorder	7 (8.3%)	2 (2.4%)	11 (8.5%)	6 (4.7%)
Posttraumatic stress disorder	5 (6.0%)	2 (2.4%)	10 (7.8%)	6 (4.7%)
Acute stress disorder	1 (1.2%)	0 (0%)	0 (0%)	
Adjustment disorder	0 (0%)		1 (0.8%)	0 (0%)
Other	1 (1.2%)	0 (0%)	0 (0%)	
Other	1 (1.2%)	1 (1.2%)	2 (1.6%)	2 (2.6%)
ADD/ADHD	1 (1.2%)	1 (1.2%)	2 (1.6%)	2 (2.6%)

*Note*: The Other category for Mood Disorders included postpartum depression, premenstrual dysphoric disorder, and seasonal affect disorder. The Other category for Anxiety Disorders included panic attacks (without panic disorder), medication‐induced anxiety, and general or unspecified anxiety.

Interviews determined that 23.8% of participants currently met criteria for BED, and an additional 13.1% reported subthreshold symptoms. With respect to NES, 8.3% of participants met diagnostic criteria, with an additional 17.9% reporting subthreshold symptoms. Based on BDI‐II scores, 33.3% of participants met the threshold for mild depression, 25.0% for moderate depression, and 6.0% for severe depression (i.e., 64.3% reported mild or greater depressive symptoms).

Use of psychiatric medications was low, with 6% of participants reporting prescription medication (selective serotonin reuptake inhibitors; SSRIs) for depression (3.6%) or anxiety (2.4%). One participant reported the use of stimulant medication for ADHD, and one participant reported the use of a sedative/hypnotic for a sleep disorder, for a total of 8.3% of participants reporting the use of psychiatric medication.

### 3.2 Study 2

Study 2 included 129 participants, who predominantly identified as white (Table 1). As in Study 1, mood disorders were the most frequently endorsed category, with 31.0% reporting a diagnosis of major depressive disorder (Table 2). Anxiety disorders were the next most frequently endorsed category, including generalized anxiety disorder and panic disorder. Trauma and stressor‐related disorders affected a similar proportion of participants as in Study 1. Rates of current diagnoses were higher in Study 2 than Study 1: 17.8% of participants reported a current mood disorder, and 16.3% reported a current anxiety disorder. One participant reported a history of a diagnosed eating disorder.

Interviews found that 18.6% of participants met criteria for BED; 20.2% reported subthreshold BED symptoms; 4.7% met criteria for NES; and 6.2% reported subthreshold NES symptoms. Based on BDI‐II scores, 18.6% of participants met the threshold for mild depression, 34.1% for moderate depression, and 1.6% for severe depression (54.3% total).

Rates of prescribed psychiatric medication were higher in Study 2 than in Study 1: 17.8% of participants reported taking at least one psychiatric medication, of which seven reported use of two or more medications. Specifically, 12.4% of participants reported taking at least one medication for anxiety (SSRIs, benzodiazepines, anxiolytics, or serotonin and norepinephrine reuptake inhibitors; SNRIs); an additional 6.2% of participants reported taking an SSRI or SNRI for depression; and one participant reported taking one SSRI for both depression and anxiety. Three participants reported the use of medications for post‐traumatic stress disorder (PTSD; SSRI, antihypertensive, or alpha‐2 receptor antagonist), of which two reported that the medication was also for depression. One participant reported the use of an SSRI for premenstrual dysphoric disorder, one participant reported taking an SNRI for ADHD, and two participants reported taking a sedative/hypnotic for insomnia.

### 3.3 Exploratory analyses

Combining samples from Studies 1 and 2 yielded a total sample size of 213 participants (Black *n* = 97, white *n* = 108, participants from other racial groups = 8). WBIS scores did not differ significantly by participant race, which may be due to the fact that all participants had elevated WBIS scores. White participants (compared to Black participants or those from other racial backgrounds) were significantly more likely to report a lifetime diagnosis of a mood (*χ* ^2^ [1, 212] = 8.421, *p* = 0.004) or anxiety disorder (*χ* ^2^ [1, 212] = 7.029, *p* = 0.008). Results were consistent when Black participants were included as the independent variable in comparison to non‐Black participants. No significant differences by race were found for full or subthreshold BED or for use of psychiatric medication.

## 4 DISCUSSION

This study is the first to report the prevalence of diagnosed psychiatric disorders among treatment‐seeking adults with obesity who have experienced and internalized weight stigma. Across two samples with diverse participants, approximately 25%–33% reported a lifetime history of a mood disorder diagnosis (predominantly major depressive disorder). Most diagnoses were not current, and use of psychiatric medications was relatively low (especially in Study 1). However, based on BDI‐II scores, 54%–64% of participants reported mild or greater symptoms of depression, with approximately 30%–35% reporting moderate to severe symptoms. This discrepancy between diagnosed and treated depression versus self‐reported symptoms requires further exploration to determine whether barriers to diagnosis/treatment may be related to weight stigma. For example, avoidance of healthcare settings due to concerns about weight stigma^5^ may reduce the likelihood of receiving a diagnosis or treatment for psychiatric concerns. In addition, doctors may prioritize weight loss recommendations if they attribute psychological distress to weight, rather than identifying depression as a psychiatric diagnosis that warrants treatment.

Anxiety disorders and PTSD affected fewer participants than mood disorders, although anxiety was the leading reason for taking psychiatric medication in Study 2. More participants in Study 2 (a predominantly white sample) endorsed a history of psychiatric diagnoses and current use of psychiatric medications than did participants in Study 1 (predominantly Black). Furthermore, exploratory analyses using data combined across samples found that white participants were more likely to report mood and anxiety disorder diagnoses than Black participants and those from other racial/ethnic minority backgrounds. This is consistent with population‐level data showing lower rates of psychiatric disorders, and of mental health care utilization, among Black versus white adults.^22^, ^23^ More research is needed to determine whether this dissimilarity reflects barriers to accessing mental health care, and/or differences in doctor‐patient interactions by race (and its intersection with weight) that affect the diagnosis and treatment of psychiatric disorders.

Prevalence of diagnosed eating disorders was low in both samples, despite a significant proportion of participants reporting full or subthreshold symptoms of BED or NES. Eating disorders in persons with a high weight may be overlooked by health professionals and patients due to stereotypes and misconceptions about both weight and eating disorders.^24^ For example, loss‐of‐control eating may be erroneously viewed as normative for individuals with a high weight, rather than being identified as an eating disorder symptom. Adults who have internalized weight stigma may be particularly susceptible to self‐blame and shame for eating disorder symptoms and may be less likely to discuss these concerns with health professionals. Future research may investigate weight self‐stigma as a barrier that prevents patients from seeking treatment for eating disorders.

Strengths of the current study include assessment by interview of past and current psychiatric disorders, medications, and diagnostic criteria for BED and NES; replication of study findings across two separate samples; and examination of a unique population of persons with high body weight who have experienced and internalized weight stigma. The main limitation of this study is the trial exclusion criteria that likely affected the prevalence of severe psychiatric disorders, current prevalence of eating disorders, and use of certain medications. Participants' self‐report of diagnoses may have yielded a lower prevalence of psychiatric disorders than if symptoms were assessed through diagnostic interview. Findings also may not generalize to adults with obesity who are not seeking weight‐loss treatment.

This work has implications for assessing and addressing the psychiatric needs of patients who report weight‐related distress and self‐stigma, and for understanding stigma‐related barriers to meeting these needs. Causal data are lacking to determine how reducing stigma may affect mental health, or how treating psychiatric disorders may affect internalized stigma. Greater attention to weight stigma in mental health research and treatment settings is needed to examine the impact of weight stigma on psychiatric symptoms and treatment outcomes. Finally, the relatively high rates of depression and anxiety in these samples (compared to previously‐reported rates among US adults with obesity)^25^ further emphasize the need for structural interventions to eradicate weight stigma and prevent its internalization.

## CONFLICT OF INTEREST STATEMENT

RLP received grant support for part of the current work, on behalf of the University of Pennsylvania, from WW International, Inc. TAW serves on advisory boards for Novo Nordisk and WW International, Inc. and has received grant support, on behalf of the University of Pennsylvania, from Epitomee Medical Co. and Novo Nordisk.
